# Severe Granulomatosis With Polyangiitis Treated With Low-Dose Cyclophosphamide, Rituximab, Glucocorticoids, and Plasma Exchange: A Case Report

**DOI:** 10.7759/cureus.64765

**Published:** 2024-07-17

**Authors:** Michael Mira, Nosagie Ohonba, Vlad Vayzband, Ryan Brown

**Affiliations:** 1 Internal Medicine, Overlook Medical Center, Summit, USA; 2 Nephrology, Overlook Medical Center, Summit, USA

**Keywords:** diffuse alveolar hemorrhage, granulomatosis with polyangiitis (gpa), gpa, nephrology, internal medicine

## Abstract

Granulomatosis with polyangiitis (GPA) is a systemic vasculitis that affects blood vessels and presents with vague constitutional symptoms, but more serious manifestations can develop, including pulmonary complications and glomerulonephritis. Currently, there are no definitive treatment guidelines. We present a case of a 66-year-old male with no previous medical history who was admitted for generalized constitutional symptoms for the past month. Imaging of the patient's brain revealed dural enhancement. Bronchoalveolar lavage was done and revealed diffuse alveolar hemorrhage (DAH). A kidney biopsy revealed granulomatosis with polyangiitis. The patient’s hospital course was complicated by acute renal failure and required hemodialysis. Due to the patient's multi-organ involvement, the patient was treated aggressively with cyclophosphamide, rituximab, plasma exchange (PE), and steroids. GPA is a systemic vasculitis that can present with multi-organ involvement. A prompt diagnosis is necessary to initiate treatment and preserve organ function. More research is needed to determine which combination therapies are the best treatment modalities in cases of severe multi-organ system involvement.

## Introduction

Granulomatosis with polyangiitis (GPA) is a necrotizing vasculitis that affects small to medium vessels and is highly associated with anti-neutrophil cytoplasmic antibodies (ANCA). GPA is characterized by necrotizing glomerulonephritis and granulomatous and necrotizing lesions that predominately affect the respiratory tract, but non-granulomatous extravascular inflammation is common [1]. The incidence of GPA is 12.8 cases per million populations per year, with the typical age of onset being 45-64 and slightly more common in males. GPA can present with life-threatening features, including rapidly progressive glomerulonephritis and diffuse alveolar hemorrhage (DAH) [2]. GPA requires prompt diagnosis, so early treatment can be initiated to manage acute symptoms and prevent long-term sequelae of ANCA-associated vasculitis. Currently, there are no standardized treatment guidelines for patients with GPA who present with life-threatening features, but the use of glucocorticoids, immunosuppressive therapy, and plasma exchange (PE) has been shown to be efficacious [3]. We report a case of GPA who developed rapidly progressive glomerulonephritis, DAH, and neurological involvement, which was treated successfully with a single dose of cyclophosphamide, rituximab, glucocorticoids, and plasma exchange.

## Case presentation

**Figure 1:**
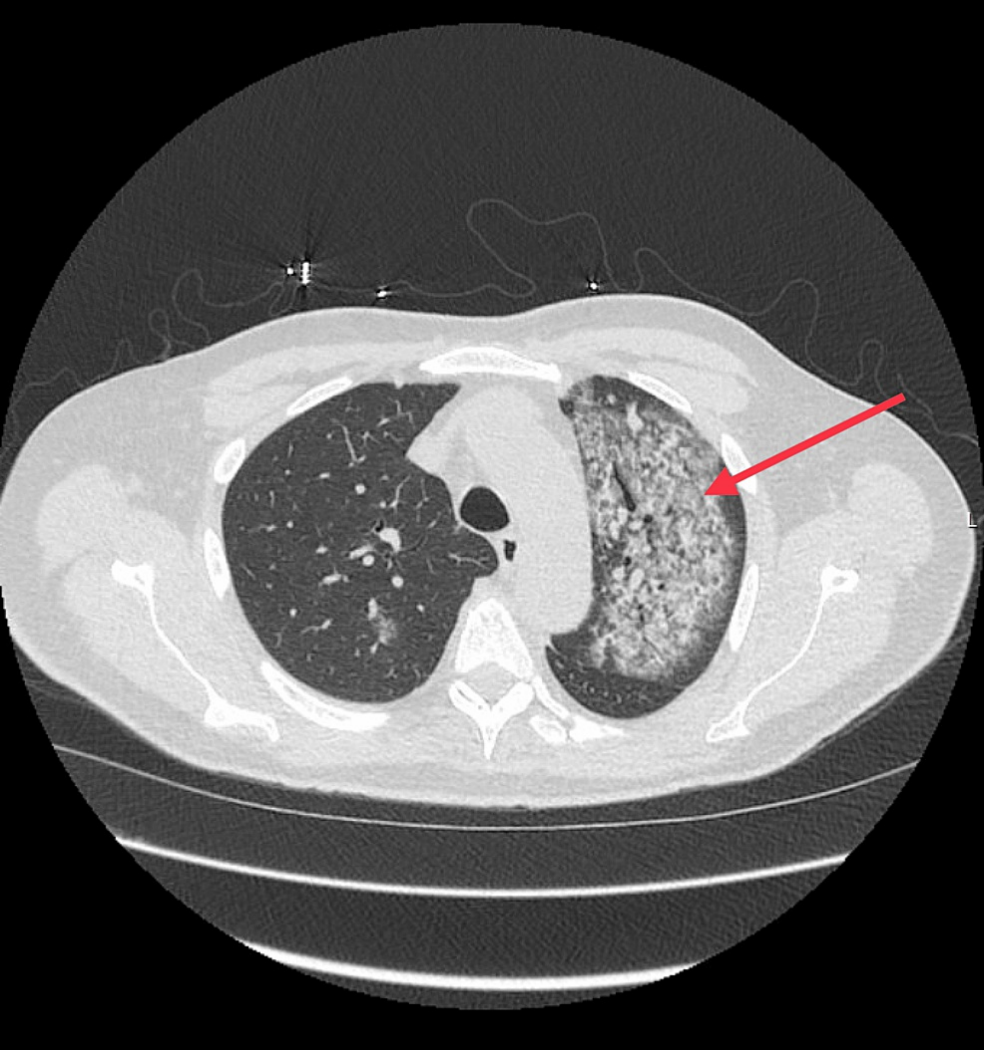
CT chest showing diffuse abnormal opacification (arrow)

A 66-year-old male with no known previous medical history was admitted for fatigue, cough, dyspnea, and 10 lb weight loss over the past month. Vital signs and physical examination were unremarkable on presentation. Laboratory tests revealed WBC 13.1 × 109 L, hemoglobin 11.9 g/dL, serum sodium 127 mmol/L, serum creatinine 0.86 mg/dL, and blood urea nitrogen (BUN) 15 mg/dL. The erythrocyte sedimentation rate was elevated to 76 mm/hr, and C-reactive protein was raised to 208 mg/dL. Urinalysis revealed moderate blood pressure and 1+ proteinuria. Urine protein excretion collected over 24 hours was 864 mg, and the urine protein/creatinine ratio was 650 mg/g. Serine protease-3 C-ANCA was highly positive at >150. Complement C3 and C4, and glomerular basement membrane antibodies were negative. Chest computed tomography (CT) (Figure 1) and chest X-rays showed bilaterally poorly defined lung lesions, and MRI brain imaging revealed dural enhancement along the convexities bilaterally.

**Figure 2:**
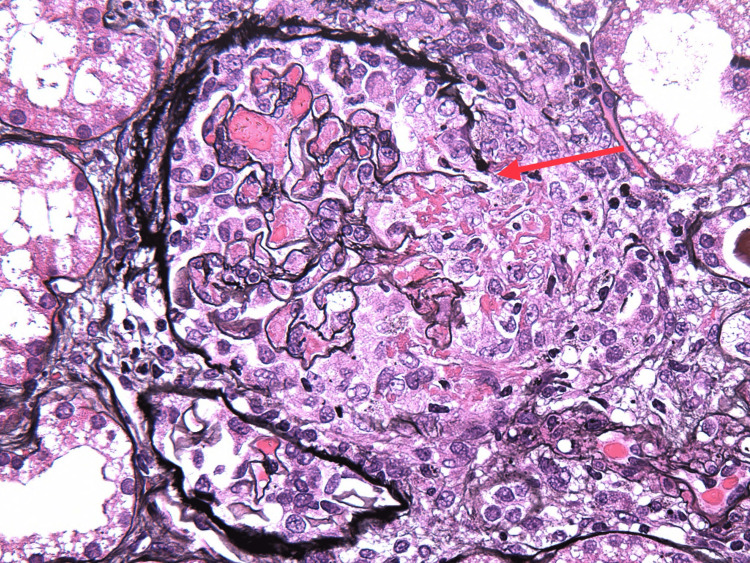
Crescent with rupture of bowman capsule (arrow)

**Figure 3:**
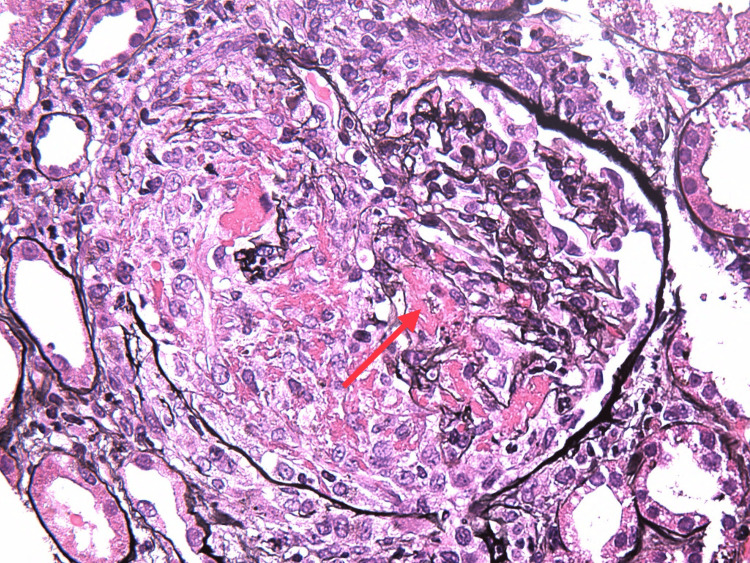
Fibrinoid necrosis (arrow) with cellular crescent

**Figure 4:**
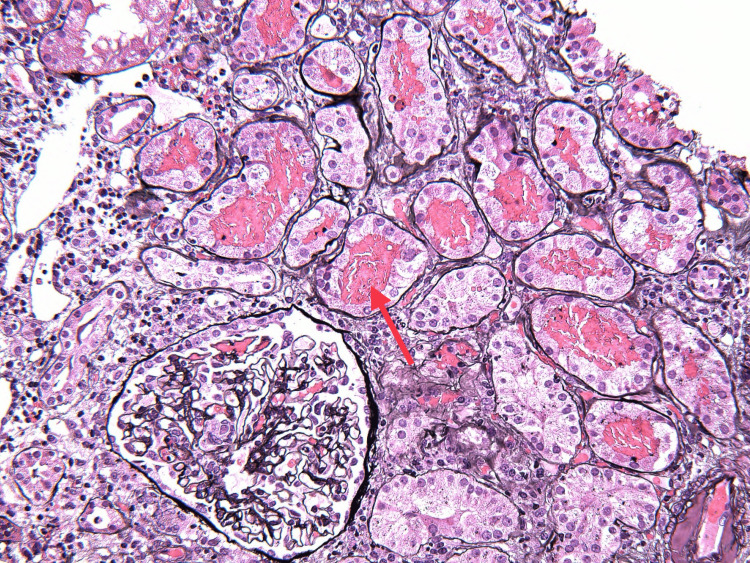
Red blood cell casts

**Figure 5:**
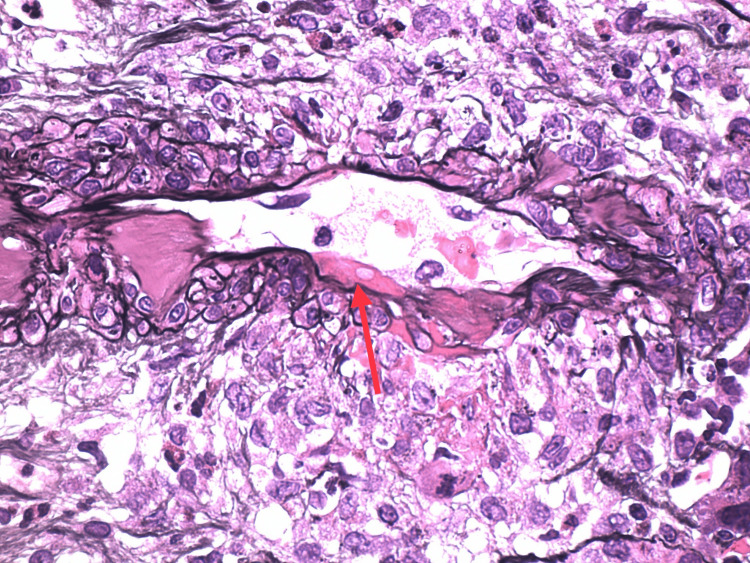
Necrotizing arteritis (arrow)

**Figure 6:**
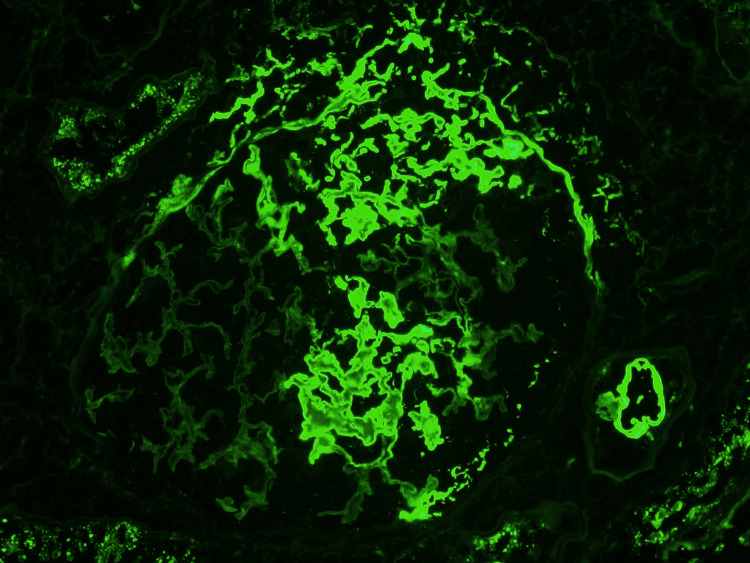
Immunofluorescence staining for fibrin highlights fibrin glomerulus

On the ninth day of admission, the patient’s creatinine rose to 3.1 mg/dL, and urinalysis revealed a large amount of blood, 2+ protein, erythrocytes, and leukocytes seen on microscopy. The patient was started on IV methylprednisone 1000 mg for three days, followed by an oral prednisone 60 mg taper, avacopan 30 mg twice daily, and prophylactic atovaquone 1500 mg daily. A kidney biopsy was performed, and histopathology revealed focal pauci-immune necrotizing and crescentic glomerulonephritis, arteritis with necrotizing and granulomatous features involving interlobular arteries, red blood cell casts, and moderate interstitial inflammation, establishing the diagnosis of granulomatous with polyangiitis (Figures 2-6). Over the following four weeks, the patient received rituximab 1000 mg, followed by three doses of rituximab 700 mg.

On the 19th day of the patient’s hospital course, his renal function continued to worsen with a creatinine of 9.6 mg/dL and BUN of 136 mg/dL, and he underwent two sessions of hemodialysis with a two-day interval. Bronchoscopy and bronchoalveolar lavage were performed, and DAH was indicated. Also, his hospital course was further complicated by left distal lower extremity weakness, paresthesias, and foot drop. Due to the severity of his multi-organ disease and his failure to improve on the current regimen, the decision was made for more aggressive treatment. He received one dose of IV cyclophosphamide (750 mg), and seven treatments of plasma exchange (PE) were performed over eight days.

After the combination treatment of cyclophosphamide, rituximab, and PE, the patient improved clinically with the resolution of his diffuse alveolar hemorrhage and recovery of his renal function. He was hospitalized for 34 days and discharged on a prednisone taper of 30 mg daily, avacopan of 30 mg twice daily, and atovaquone 1500 mg daily.

## Discussion

GPA, previously known as Wegener's granulomatosis, presents intricate diagnostic and therapeutic challenges as a complex autoimmune vasculitis characterized by necrotizing inflammation of small to medium-sized blood vessels with multi-organ involvement. The case of this 66-year-old male patient underscores the diverse and nonspecific clinical presentation of GPA. Initial manifestations can include fatigue, cough, dyspnea, and weight loss. These symptoms can mimic common respiratory or systemic illnesses, necessitating a high index of suspicion of vasculitis for an accurate diagnosis.

Genetic, epigenetic, and environmental factors contribute to the etiology and pathogenesis of ANCA-associated vasculitis (AAV). Different triggers can lead to the production of autoantibodies (ANCA) that, in the context of an inflammatory environment, can cause tissue inflammation and vascular injury, which have been proposed from several different mechanisms such as alternations in HLA-mediated presentation, T and B lymphocyte activation, defective immune regulation, and atypical antigen structure or function [4].

Laboratory investigations play a crucial role in the diagnostic workup of GPA, with elevated inflammatory markers such as erythrocyte sedimentation rate (ESR) and C-reactive protein (CRP), alongside serological findings of positive serine protease-3 C-ANCA and myeloperoxidase, aiding in the confirmation of the diagnosis. A large international cohort study by Kronbichler et al. showed several clinical and laboratory factors were identified as "more" associated with renal involvement in ANCA-associated vasculitis, which included fatigue, weight loss, high C-reactive protein, low complement 3, myeloperoxidase, and proteinase 3-ANCA [5].

Renal involvement, seen in up to 70-85% of GPA patients, is a hallmark feature, often presenting as focal segmental glomerulonephritis (GN) or crescentic rapidly progressive GN (RPGN). Idolor et al. suggested there was no statistically significant difference in inpatient mortality for hospitalizations of GPA with and without renal involvement [6]. The renal biopsy findings in this case, demonstrating pauci-immune necrotizing and crescentic glomerulonephritis with arteritis, highlight the histopathological complexity of renal manifestations in GPA, which is essential for accurate diagnosis and treatment planning.

Treatment strategies for GPA focus on inducing remission, alleviating systemic inflammation, and preserving organ function. Standard therapy typically includes immunosuppressive drugs like cyclophosphamide or rituximab in combination with glucocorticoids. Rituximab demonstrates comparable efficacy to cyclophosphamide in initiating remission, while proving more efficacious in cases of relapse or refractory disease. The recommendation favors combining low-dose glucocorticoids with milder immunosuppressants like azathioprine, rituximab, or methotrexate instead of cyclophosphamide [7]. The KDIGO 2021 guidelines for the treatment of AAV advise patients presenting with a greatly reduced or rapidly declining GFR (serum creatinine > 4 mg/dL) that cyclophosphamide and glucocorticoids are preferred for induction therapy. However, a combination of rituximab and cyclophosphamide can also be used. Plasma exchange can be considered in more severe cases of AAV in patients who are oliguric, have serum creatinine >5.7 mg/dL, or have alveolar hemorrhage with hypoxemia, where mortality is high. The guidelines recommend maintenance therapy with either rituximab or azathioprine and low-dose glucocorticoids after induction of remission [8]. In refractory cases or when standard therapy is unsuitable, intravenous immunoglobulins (IVIG) may be considered, although optimal dosing remains uncertain. The ACR 2021 guidelines for AAV management suggest considering the addition of IVIG as a supplementary treatment in individuals with GPA or MPA who cannot undergo other immunomodulatory therapies. This recommendation also extends to patients receiving rituximab for remission maintenance, particularly if they have hypogammaglobulinemia and experience recurrent severe infections [9]. Lastly, plasmapheresis, recommended by the American Society of Apheresis for severe cases, particularly in dialysis-dependent patients, serves as a second-line treatment option, alongside other therapeutic modalities, to manage the severity and context of the disease.

In the presented case, the patient's worsening renal function and multi-organ involvement necessitated a more aggressive treatment approach, including low-dose cyclophosphamide, rituximab, and PE. Following this combination therapy, the patient showed clinical improvement with resolution of diffuse alveolar hemorrhage and recovery of renal function, highlighting the importance of tailored and multidisciplinary management in achieving favorable outcomes in GPA patients.

## Conclusions

Effective management of GPA requires a multidisciplinary approach involving careful clinical assessment, laboratory investigations, and histopathological evaluation. In this case, the diagnosis was confirmed through serological findings and a renal biopsy, highlighting the importance of a comprehensive diagnostic workup in guiding therapeutic decisions.

Treatment strategies for GPA aim to induce remission, alleviate inflammation, and preserve organ function. Standard therapies, including immunosuppressive drugs like cyclophosphamide and/or rituximab and glucocorticoids, remain the cornerstone of treatments. However, in severe cases, combination therapy with multiple agents and plasma exchange may be necessary to achieve favorable outcomes. This case emphasizes the need for early recognition, prompt intervention, and individualized treatment strategies in the management of GPA, thereby improving patient outcomes and quality of life.
